# Three-dimensional CBCT evaluation of nasal and pharyngeal airway changes after rapid maxillary expansion: A study among growing children

**DOI:** 10.6026/973206300221944

**Published:** 2026-04-30

**Authors:** Hiba Ikhlas Zain, B.L Prasanna Thathapudi, Mandar Nandkishor Pathak, Deekshith Shetty, Thejaswini Nallamari, Dwijesh S Goswami

**Affiliations:** 1 Department of Orthodontist, Moses Dental Clinic, Guruvayur, Thrissur, Kerala-680101, India; 2 Department of Orthodontics and Dentofacial, Sree Sai Dental College, Ranasthali, New Colony, Srikakulam Andhra Pradesh-532001, India; 3 Department of Orthodontics, SDDC Parbhani, Parawa, Maharashtra 431402, India; 4 Department of Orthodontics, KVG Dental College and Hospital, Kurunjibagh, Sullia, Karnataka 574327, India; 5 Department of Orthodontics and Dentofacial Orthopedics, CKS Theja Institute of Dental sciences and research, Tirupati, Andhra Pradesh 517506, India; 6 Department of Orthodontics and Dentofacial Orthopaedics, Ahmedabad Dental College and Hospital, Bhadaj - Ranchodpura Road, Sardar Patel Ring, Ahmedabad, Gujarat 382115, India

**Keywords:** Rapid maxillary expansion (RME), cone-beam computed tomography (CBCT), upper airway, nasal cavity, pharyngeal airway, pediatric orthodontics, airway volume

## Abstract

Maxillary transverse deficiency in growing children is associated with compromised nasal airway dimensions and altered respiratory function. Therefore, it is of interest to evaluate three-dimensional nasal and pharyngeal airway changes in 68 children aged 8-14 years before and six months after rapid maxillary expansion. Significant increases were observed in total nasal cavity volume (26.0%, p<0.001), minimum nasal cross-sectional area (34.2%, p<0.001) and nasopharyngeal volume (20.0%, p<0.001), while oropharyngeal changes were not statistically significant (p>0.05). The magnitude of nasal volumetric gain showed moderate correlation with amount of skeletal expansion achieved (r=0.542, p<0.001). Thus, rapid maxillary expansion produces substantial and region-specific upper airway enlargement, primarily affecting nasal and nasopharyngeal compartments in growing patients.

## Background:

Maxillary transverse deficiency is a common craniofacial condition in growing children and is frequently associated with posterior crossbite and reduced nasal airway dimensions [1]. Impaired transverse maxillary development has been linked to increased nasal resistance and altered breathing patterns, including mouth breathing [2]. Rapid maxillary expansion (RME) is an established orthopedic procedure that separates the mid-palatal suture to correct transverse discrepancies during growth [3]. Beyond dental correction, RME induces skeletal changes that may influence adjacent nasal and pharyngeal airway structures [4]. The anatomical relationship between the maxilla and nasal cavity floor suggests that lateral maxillary displacement increases nasal width and potentially airway volume [5]. Recent three-dimensional imaging studies have demonstrated measurable increases in nasal cavity dimensions following RME [6]. CBCT-based volumetric analysis allows accurate assessment of airway changes with minimal superimposition error compared to two-dimensional imaging [7]. This technique provides reliable evaluation of volumetric, linear and cross-sectional airway parameters in growing patients [8]. While several studies report nasal airway enlargement after RME, findings regarding pharyngeal airway response remain inconsistent [9]. Some investigations demonstrate nasopharyngeal volume increases, whereas oropharyngeal changes are variable and often non-significant [10]. Differences in sample age, skeletal maturity, follow-up duration and segmentation protocols contribute to heterogeneity in reported outcomes [11]. Standardized CBCT protocols and adequate follow-up are required to clarify the regional airway response to expansion in growing populations. Understanding the magnitude and distribution of airway changes is clinically relevant in the context of pediatric airway management and sleep-disordered breathing [12]. However, high-quality prospective studies with uniform methodology remain limited. Therefore, it is of interest to report three-dimensional nasal and pharyngeal airway changes following rapid maxillary expansion in growing children using standardized CBCT analysis.

## Materials and Methods:

This prospective longitudinal study was conducted in the Department of Orthodontics after obtaining institutional ethical approval. Sample size was calculated using G*Power version 3.1.9.4 based on detection of a minimum clinically significant difference of 1,500 mm^3^ in nasal cavity volume with a standard deviation of 2,000 mm^3^, power of 0.85 and alpha of 0.05, resulting in a minimum requirement of 58 participants; considering a 15% attrition rate, 68 participants were recruited. Children aged 8-14 years presenting with maxillary transverse deficiency of ≥4 mm and unilateral or bilateral posterior crossbite were included. All participants were in mixed or early permanent dentition and at cervical vertebral maturation stages CS1-CS3. Patients with craniofacial syndromes, cleft lip or palate, recent adenoidectomy or tonsillectomy, severe skeletal discrepancies, active respiratory infection, or poor-quality CBCT scans were excluded. All patients were treated using bonded Hyrax-type rapid palatal expanders with acrylic coverage of posterior teeth. Initial activation was 0.5 mm, followed by 0.5 mm per day until adequate overcorrection was achieved, typically within 7-14 days. The expander remained as a passive retainer for six months. CBCT scans were obtained at baseline (T0) within one week prior to expansion and at six months post-expansion (T1). Imaging was performed using the same CBCT unit with standardized parameters including 17 x 23 cm field of view; 0.3 mm voxel size, 90 kVp, 10 mA and 12-second scan time. Patients were positioned in natural head position with Frankfort horizontal plane parallel to the floor, teeth in maximum intercuspation and tongue resting against the palate. Scans were acquired during afternoon hours to minimize circadian variation. CBCT data were exported in DICOM format and analyzed using Dolphin Imaging 3D software version 11.95. Airway spaces were semi- automatically segmented using threshold-based techniques with manual refinement. Anatomical boundaries for nasal cavity, nasopharynx and oropharynx were predefined according to cranial base, palatal plane, choanal and epiglottic landmarks. Volumetric measurements included total nasal cavity, nasopharyngeal, oropharyngeal and total pharyngeal airway volumes. Linear measurements included nasal cavity widths at superior, middle and inferior levels and anteroposterior depths of pharyngeal segments. Cross-sectional parameters included minimum and average cross-sectional areas of nasal and pharyngeal compartments. Intra-examiner reliability was assessed by re-evaluating 20 randomly selected scans after four weeks and intraclass correlation coefficients demonstrated excellent agreement. Statistical analysis was performed using SPSS version 27.0, with normality assessed using Shapiro-Wilk tests. Paired comparisons between T0 and T1 were performed using appropriate parametric tests and correlation analysis was conducted to evaluate the relationship between expansion magnitude and airway changes, with significance set at p<0.05.

## Results:

A total of 68 growing patients completed the study with no attrition. The mean age was 10.8 ± 1.6 years, with balanced sex distribution. The mean maxillary expansion achieved was 7.2 ± 1.4 mm over an average duration of 10.4 ± 2.6 days. All participants tolerated the procedure without adverse complications. Significant increases were observed in all nasal cavity volumetric parameters at six months post-expansion. Total nasal cavity volume increased by 2,815.8 mm^3^, representing a 26.0% gain (p<0.001) with a large effect size (Cohen's d=1.18). Bilateral nasal cavity volumes increased symmetrically, each demonstrating approximately 26% enlargement. Linear nasal width measurements showed statistically significant increases at superior, middle and inferior levels, with the greatest increase at the inferior level (3.8 mm). Minimum nasal cross-sectional area increased by 34.2% (p<0.001), indicating substantial airway enlargement at the most constricted region. Nasal cavity height did not demonstrate significant change (p=0.724), suggesting predominantly transverse expansion. Nasopharyngeal airway volume increased by 20.0% (p<0.001), accompanied by significant increases in anteroposterior depth and lateral width. In contrast, oropharyngeal volumetric and dimensional changes were minimal and statistically non-significant (p>0.05). Total pharyngeal airway volume increased primarily due to nasopharyngeal enlargement. Subgroup analysis revealed no significant differences in airway response between younger and older participants or between males and females. Correlation analysis demonstrated a moderate positive relationship between amount of skeletal expansion and nasal cavity volumetric increase (r=0.542, p<0.001). No significant correlation was observed between expansion magnitude and oropharyngeal changes. These findings indicate region-specific airway response to rapid maxillary expansion in growing children. Table 1 (see PDF) shows that the study included 68 participants with a mean age of 10.8 ± 1.6 years, 41.2% aged 8-10 years and 58.8% aged 11-14 years, with a nearly balanced sex distribution of 47.1% males and 52.9% females, mean BMI of 18.4 ± 3.2 kg/m^2^, unilateral crossbite present in 61.8% and bilateral crossbite in 38.2%, mean maxillary width deficiency of 5.8 ± 1.2 mm, cervical vertebral maturation stages distributed as CS1 (26.5%), CS2 (47.1%) and CS3 (26.5%), mean expansion achieved of 7.2 ± 1.4 mm over 10.4 ± 2.6 days, with 50.0% reporting mouth breathing and 23.5% having allergic rhinitis. Table 2 (see PDF) demonstrates that total nasal cavity volume increased from 10,842.6 ± 2,346.8 mm^3^ to 13,658.4 ± 2,542.1 mm^3^, representing a 26.0% increase (p<0.001, Cohen's d=1.18), with right and left nasal cavity volumes increasing symmetrically by approximately 26%, nasal width increasing significantly at the superior (9.7%), middle (15.0%) and inferior (15.3%) levels, the greatest absolute increase observed at the inferior level (3.8 mm), minimum nasal cross-sectional area increasing from 154.6 ± 42.8 mm^2^ to 207.4 ± 48.2 mm^2^ corresponding to a 34.2% increase (p<0.001), average nasal cross-sectional area increasing by 25.1% and nasal cavity height showing no significant change (0.5%, p=0.724). Table 3 (see PDF) indicates that nasopharyngeal volume increased from 4,286.3 ± 1,124.5 mm^3^ to 5,142.7 ± 1,268.4 mm^3^, reflecting a 20.0% increase (p<0.001, Cohen's d=0.72), total pharyngeal volume increased by 8.5% (p<0.001), nasopharyngeal anteroposterior depth and lateral width increased significantly by 8.3% and 9.8% respectively, minimum nasopharyngeal cross-sectional area increased by 22.7% (p<0.001), while oropharyngeal volume increased by only 2.8% (p=0.142) with non- significant changes in anteroposterior depth, lateral width and cross-sectional areas.

## Discussion:

This prospective CBCT investigation demonstrates that rapid maxillary expansion produces substantial and region-specific enlargement of the upper airway in growing children. The 26.0% increase in total nasal cavity volume and 34.2% rise in minimum nasal cross-sectional area indicate clinically meaningful transverse airway expansion. Recent CBCT-based studies have similarly reported significant nasal volumetric gains following RME in growing patients, supporting the reproducibility of skeletal-airway coupling during active sutural growth [13, 14]. The large effect size observed in nasal volume change reinforces the magnitude of orthopedic influence on nasal architecture. The anatomical basis of these findings relates to separation of the mid-palatal suture and lateral displacement of maxillary halves, which form the floor and lateral walls of the nasal cavity [15]. The greater width increases at the inferior nasal level compared to the superior level reflects the pyramidal expansion pattern described in contemporary imaging studies [16]. The absence of significant change in nasal height confirms that the effect is predominantly transverse rather than vertical. Increased minimum nasal cross-sectional area is particularly relevant because small dimensional changes at the narrowest airway region can significantly influence airflow resistance and nasal patency [17]. Nasopharyngeal volume increased by 20.0%, while oropharyngeal dimensions remained largely unchanged. Similar region-specific responses have been described in recent longitudinal CBCT analyses, where nasopharyngeal enlargement was attributed to its anatomical proximity to the posterior maxilla and circummaxillary sutural system [18]. In contrast, the oropharynx is influenced by soft tissue structures including tongue posture and soft palate position, which may explain its relative resistance to skeletal expansion effects. The moderate effect size in nasopharyngeal changes supports a biologically meaningful but less pronounced response compared to the nasal cavity [19]. The moderate positive correlation between amount of expansion and nasal volumetric gain indicates a dose-response relationship between skeletal displacement and airway enlargement. This association has been observed in recent quantitative CBCT investigations, emphasizing that greater transverse skeletal correction yields proportionally greater nasal airway improvement [20]. The absence of correlation with oropharyngeal changes further confirms the localized biomechanical influence of RME.

## Conclusion:

Rapid maxillary expansion produces significant and region-specific enlargement of the nasal cavity and nasopharyngeal airway in growing children, with minimal impact on the oropharynx. The magnitude of nasal volumetric gain and increase in minimum cross-sectional area indicate clinically meaningful improvement in airway dimensions sustained at six months post-expansion. Thus, we show the role of RME as a skeletal intervention with adjunctive airway benefits, while highlighting the need for long-term functional studies to confirm respiratory implications.

## Advancement to knowledge:

Advancement to knowledge in this study lies in the standardized prospective design, six-month retention-based follow-up and comprehensive segmentation of nasal and pharyngeal compartments using uniform CBCT parameters. Unlike cross-sectional or short-term analyses, the present findings demonstrate sustained airway enlargement after completion of retention, strengthening evidence for stability of nasal and nasopharyngeal changes during growth. The integration of volumetric, linear and cross-sectional metrics provides a multidimensional understanding of airway adaptation rather than relying solely on volume assessment. Clinically, these findings support the concept that RME offers airway benefits beyond correction of transverse maxillary deficiency. However, RME should not be considered a standalone therapy for pediatric sleep-disordered breathing without multidisciplinary evaluation. Future studies incorporating objective respiratory function tests and long-term follow-up into adolescence are necessary to determine the durability and functional translation of these anatomical changes.
